# Community Prescribing and Resistant *Streptococcus pneumoniae*

**DOI:** 10.3201/eid1106.050198

**Published:** 2005-06

**Authors:** Galia Barkai, David Greenberg, Noga Givon-Lavi, Eli Dreifuss, Daniel Vardy, Ron Dagan

**Affiliations:** * Soroka University Medical Center and Ben-Gurion University of the Negev, Beer-Sheva, Israel;; † Israel General Health Insurance Plan, Beer-Sheva, Israel

**Keywords:** antibiotics, antibiotic resistance, Streptococcus pneumoniae

## Abstract

We investigated the association between prescribing antimicrobial agents and antimicrobial resistance of *Streptococcus pneumoniae* among children with acute otitis media in southern Israel. During a 6-year period, all prescriptions of a sample of ≈20% of Jewish and Bedouin children <5 years of age were recorded and all pneumococcal isolates from middle ear fluid were collected. Although antimicrobial drug use was significantly higher in Bedouin children, the proportion of *S. pneumoniae* isolates with penicillin MIC ≥1.0 μg/mL was significantly higher in Jewish children. In both populations, antimicrobial prescriptions were markedly reduced over time, especially for penicillins and erythromycin. In contrast, azithromycin prescriptions increased from 1998 to 2001 with a parallel increase in macrolide and multidrug resistance. Penicillin resistance was associated with macrolide resistance. These findings strongly suggest that azithromycin affects increased antimicrobial resistance, including multidrug resistance, in *S. pneumoniae*.

Community-acquired respiratory infections are the main reason for prescribing antimicrobial agents in young children (1). Antimicrobial drug use is a major contributor to the emergence of resistance in respiratory pathogens (2–5). Rates of antimicrobial drug use vary between countries, leading to different rates of antimicrobial-resistant pathogens (6,7). Selection for resistant organisms also depends on the class of antimicrobial agent and pharmacokinetic and pharmacodynamic characteristics of the drug (2,3,7,8).

Acute otitis media is the most common microbial respiratory tract infection in early childhood and a leading reason for antimicrobial drug use in children in most industrialized countries (9). *Streptococcus pneumoniae* and *Haemophilus influenzae* are the 2 most common pathogens isolated from patients with acute otitis media worldwide, comprising >75% of all microbial episodes (10). During the last decade, an alarming increase in resistance of *S. pneumoniae* to many antimicrobial agents has been observed (11–14). In contrast, the prevalence of β-lactamase production in *H. influenzae* is relatively stable.

The dynamics of the relationship between antimicrobial drug use and prevalence of resistance in *S. pneumoniae* has not been systemically studied in children. We attempted to determine the association between prescribing antimicrobial agents and resistance patterns of *S. pneumoniae* recovered from middle ear fluid of children with acute otitis media in southern Israel over a 6-year period. We hypothesized that 1) the dynamics of antimicrobial resistance patterns of *S. pneumoniae* causing acute otitis media are associated with that of prescribing antimicrobial agents, and 2) not all antimicrobial classes contribute equally to resistance in this pathogen.

## Materials and Methods

### Setting

In southern Israel (the Negev), Jewish and Bedouin populations live side by side; the socioeconomic conditions and the lifestyles of the 2 groups differ, but both have access to the same medical services (both clinic and hospitalization services). The Jewish population is mainly urban, whereas the Bedouin population, formerly composed of desert nomads, is in transition to a western lifestyle (15). The 2 pediatric populations also differ in disease patterns and rates. Hospitalization rates for respiratory and other infectious diseases are higher among Bedouin children (16,17). The only medical center providing hospital services to the Negev is the Soroka University Medical Center, in which all children in the area are born and treated.

Medical insurance in Israel is universal and provided free of charge. Approximately 60% of Jewish children and 85%–90% of Bedouin children in the Negev are insured in the largest health plan in Israel, the General Health Insurance Plan. Antimicrobial treatment policies are identical in the 2 populations since drug formulations, prices, and availability of antimicrobial agents are the same at all General Health Insurance Plan clinics. Criteria for referral to a hospital are also similar, and no change in policy occurred during the study period regarding referral to a pediatric emergency room with a recommendation to perform tympanocentesis.

The 7-valent pneumococcal conjugate vaccine (Prevenar, Wyeth-Lederle Vaccines, Pearl River, NY, USA) has been licensed in Israel since 2004, but has not yet been introduced into the country because of a supply shortage. Thus, none of the children were vaccinated during the study.

### Measuring Antimicrobial Drug Prescriptions

In the Negev region, 53 primary urban General Health Insurance Plan clinics each care for a minimum of 3,000 members. Data were gathered from the 7 largest pediatric primary care clinics where all prescriptions were computerized during all study years. Five clinics, caring for a yearly average population of 6,163 children <5 years age, were located in urban Jewish centers; 2 clinics, caring for a yearly average population of 6,636 children <5 years of age, were located in Bedouin towns. This accounts for ≈20% of the region's children in this age group. Twenty-five to 30 physicians treated children at these clinics. All computerized data regarding prescriptions were generated from the economic department of the General Health Insurance Plan in southern Israel. All antimicrobial drug prescriptions for outpatient children <5 years of age were recorded from 1998 through 2003 and grouped as follows: penicillins (amoxicillin, amoxicillin-clavulanate, and phenoxymethyl penicillin); cephalosporins (cefaclor, cephalexin monohydrate, and cefuroxime-axetil); erythromycin (stearate or ethyl-succinate); and azithromycin. Internal data analysis showed no differences in prescribing antimicrobial agents between the different clinics within each ethnic group.

Microbial Isolates

All middle ear fluid specimens from patients treated at the Soroka University Medical Center pediatric emergency room and patients hospitalized with acute otitis media, and >90% of the middle ear fluid specimens obtained by otolaryngologists in the community, are sent for culture to the Clinical Microbiology Laboratory of this medical center, the only microbiology laboratory in the Negev. Two thirds of the aspirates were obtained at this medical center (40% in the emergency room and another 26% in pediatric wards). Patients at this center with acute otitis media and bulging tympanic membrane undergo tympanocentesis, which is done by an otolaryngologist. In the outpatient clinics (approximately one third of the patients), tympanocentesis is typically carried out in patients with recurrent or nonresponsive acute otitis media. A small proportion of middle ear fluid aspirates were obtained as part of double tympanocentesis studies. All *S. pneumoniae* isolates obtained from the middle ear fluid of children with episodes of acute otitis media from 1999 through 2003 that were processed at Soroka University Medical Center were included.

An episode was defined as a pathogen-free interval of ≥30 days between isolations for the same serotype or by any interval for different serotypes. Only 1 isolate was counted per episode; if the same strain was isolated from both ears, 1 of the 2 was randomly selected.

### Microbiologic Analysis

Swabs were placed in MW173 Amies transport medium (Transwab, Medical Wire and Equipment, Potley, UK), plated immediately on trypticase agar media containing 5% sheep blood and 5 μg/mL of gentamicin, and incubated aerobically at 35°C for 48 h. Presumptive identification of *S. pneumoniae* was based on the presence of α-hemolysis and inhibition by optochin and was confirmed by slide agglutination (Phadebact, Pharmacia Diagnostics, Uppsala, Sweden). Serotyping was conducted by using the quellung reaction with reagents from Statens Serum Institut (Copenhagen, Denmark) (18). Susceptibility of *S. pneumoniae* to sulfamethoxazole, tetracycline, erythromycin, clindamycin, and chloramphenicol was determined by using the Kirby-Bauer disk diffusion method and interpreted according to the National Committee for Clinical Laboratory Standards (19). Macrolide resistance was reported as erythromycin resistance. Isolates exhibiting inhibition zones ≤19 mm with a 1-μg oxacillin disk were further tested by Etest (PDM Epsilometer, AB Biodisk, Solna, Sweden) for penicillin (20). Isolates with a penicillin MIC ≥0.1 μg/mL were considered nonsusceptible to penicillin. Isolates with a penicillin MIC ≥1.0 μg/mL were analyzed separately. Isolates resistant to ≥3 antimicrobial classes were considered multidrug resistant.

### Statistical Analysis

Statistical analysis was done with SPSS (Chicago, IL, USA) 12.0 software for Windows. The chi-square test was used to compare the distribution of categoric data. Linear regression analysis with the Pearson correlation coefficient was done to test the linear increase or decrease of antimicrobial prescription rates. A similar procedure was performed for exponential increases or decreases in prescription rates, but variables were tested after logarithmic transformation. Comparisons of mean yearly differences in antimicrobial prescription rates between Bedouin and Jewish children were done using the paired samples *t* test. A p value <0.05 was considered significant.

## Results

### Prescribing of Antimicrobial Agents

A total of 236,466 prescriptions (149,589 for Bedouin and 86,877 for Jewish children) were recorded in the 7 clinics, which represented 12,799 child-years in children <5 years of age (Table). Overall prescribing of antimicrobial agents to Bedouin children was higher (mean ± SD 3.79 ± 0.4 prescriptions per child-year) than to Jewish children (2.37 ± 0.3 prescriptions per child-year). This represents a mean difference of 1.41 prescriptions per child-year (p<0.001).

**Table:** Antimicrobial drug prescriptions among Bedouin and Jewish children <5 years of age, southern Israel, 1998–2003

Antimicrobial agent	Bedouin children		Jewish children			
	Total prescriptions	Mean ± SD yearly prescription rate/1,000 children	Total prescriptions	Mean ± SD yearly prescription rate/1,000 children	Mean ± SD yearly difference (Bedouin children vs. Jewish children)/prescription/1,000 children	p value
Amoxicillin	89,598	2,269 ± 266	54,090	1,480 ± 264	789 ± 175	<0.001
Amoxicillin-clavulanate	27,729	703 ± 91	12,173	335 ± 88	368 ± 51	<0.001
Phenoxymethyl penicillin	3,187	84 ± 47	3,609	101 ± 47	–16 ± 10	0.012
Cephalosporins*	18,625	471 ± 69	5,751	155 ± 34	316 ± 66	<0.001
Erythromycin	3,492	92 ± 51	2,256	64 ± 45	28 ± 66	0.002
Azithromycin	6,958	167 ± 108	8,998	236 ± 124	–70 ± 49	0.018
Total	149,589	3,787 ± 442	86,877	2,371 ± 315	1,416 ± 224	<0.001

*Cefaclor, cephalexin monohydrate, and cefuroxime-axetil.

**Figure 1:**
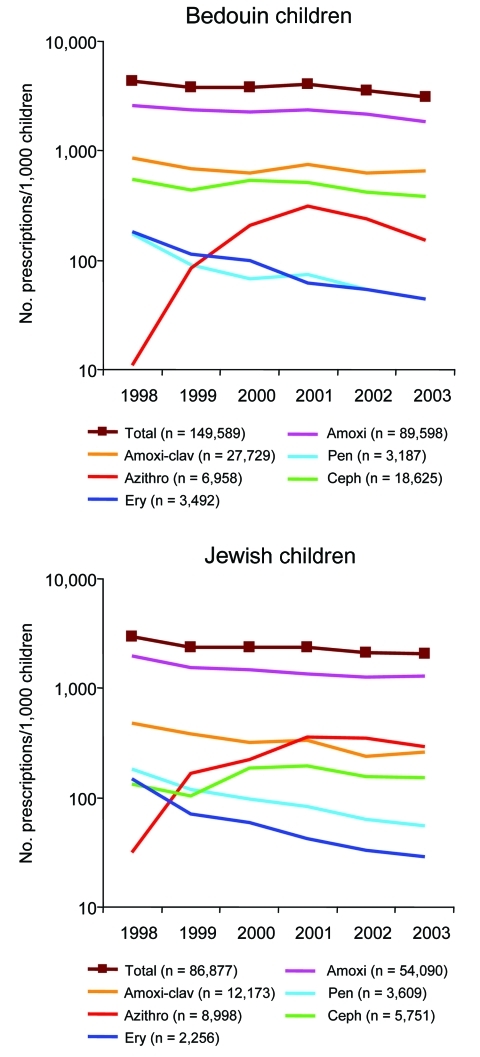
Antimicrobial drug prescription rates for Bedouin and Jewish children <5 years of age in southern Israel from 1998 through 2003. Amoxi, amoxicillin; Amoxi-clav, amoxicillin-clavulanate; Pen, phenoxymethyl penicillin; Azithro, azithromycin; Ceph, cephalosporins (cefazolin, cefaclor, cephalexin monohydrate, and cefuroxime-axetil); Ery, erythromycin.

As a group, penicillins were the most frequently prescribed agents and accounted for 80.6% (120,514/149,589) of prescriptions for Bedouin and 80.4% (69,872/86,877) for Jewish children (Table and Figure 1). The cephalosporin group ranked second in Bedouin children (12.5% of all prescriptions), and azithromycin ranked second in Jewish children (10.4% of all prescriptions). Significantly more amoxicillin (1.53-fold), amoxicillin-clavulanate (2.10-fold), cephalosporins (3.04-fold), and erythromycin (1.44-fold) were prescribed for Bedouin than for Jewish children. Jewish children received more prescriptions for phenoxymethyl penicillin (1.20-fold) and azithromycin (1.41-fold) than Bedouin children. All differences between prescription rates of Bedouin and Jewish children were significant (Table).

A significant decrease in total antimicrobial prescriptions was noted with time (Figure 1). In Bedouin children, total antimicrobial prescription rate was reduced 29% from 4,389 per 1,000 children in 1998 to 3,106 per 1,000 children in 2003 (p = 0.038, r = –0.835 for exponential decrease). In Jewish children, total prescription rate was reduced 30% from 2,949 per 1,000 children in 1998 to 2,079 per 1,000 children in 2003 (p = 0.014, r = –0.902 for exponential decrease). The decrease in total antimicrobial prescription rate in both populations was caused mostly by a reduction in the prescription rate of penicillins. The erythromycin prescription rate decreased sharply in both populations. In Bedouin children, the rate was reduced 76% from 181 prescriptions per 1,000 children in 1998 to 44 per 1,000 children in 2003 (p<0.001, r = –0.984 for exponential decrease); in Jewish children, the rate was reduced 81% from 149 prescriptions per 1,000 children in 1998 to 29 per 1,000 children in 2003 (p = 0.002; r = –0.963 for exponential decrease).

Azithromycin was introduced in Israel in 1998. The prescription rate of this drug increased dramatically to 310 prescriptions per 1,000 Bedouin children (p = 0.04, r = 0.996 for linear increase) and 357 prescriptions per 1,000 Jewish children (p = 0.011, r = 0.989 for linear increase) in 2001. Thereafter, a sharp decrease in azithromycin prescription rate (a 51% decrease to 152/1,000) was noted in Bedouin children (p = 0.024, r = –0.999 for linear decrease from 2001 to 2003). In Jewish children, the azithromycin prescription rate decreased 31%, but this did not reach statistical significance (p = not significant for linear decrease from 2001 to 2003). No significant change in the cephalosporin prescription rate was observed in these populations.

### Acute Otitis Media Episodes

A total of 11,311 episodes of acute otitis media requiring tympanocentesis were recorded in the region during the study period. At least 1 pathogen was isolated in 59% (6,678/11,311) of the episodes.

### *S. pneumoniae* Acute Otitis Media Episodes

A total of 3,651 *S. pneumoniae* acute otitis media episodes were recorded: 39% (1,425/3,651) of the isolates were obtained from Jewish children and 61% (2,223/3,651) from Bedouin children. The ethnicity of the child was not recorded for 3 isolates. In 89% (3,258/3,651) of the children, a complete history regarding previous episodes of acute otitis media and antimicrobial treatment was available. Twenty-nine percent (978/3,396) of the children had had 1–3 episodes of acute otitis media and 36% (1,225/3,396) had >3 episodes of acute otitis media in the preceding year. Twenty-five percent (823/3,258) of the episodes occurred in children who had already undergone tympanocentesis. A total of 24% (809/3,373) of the children were receiving antimicrobial agents at the time of tympanocentesis, and 36% (1,231/3,373) had received antimicrobial agents in the preceding month. No major differences in the proportions of these characteristics were seen within each ethnic group.

### *S. pneumoniae* Antimicrobial Resistance Patterns

**Figure 2:**
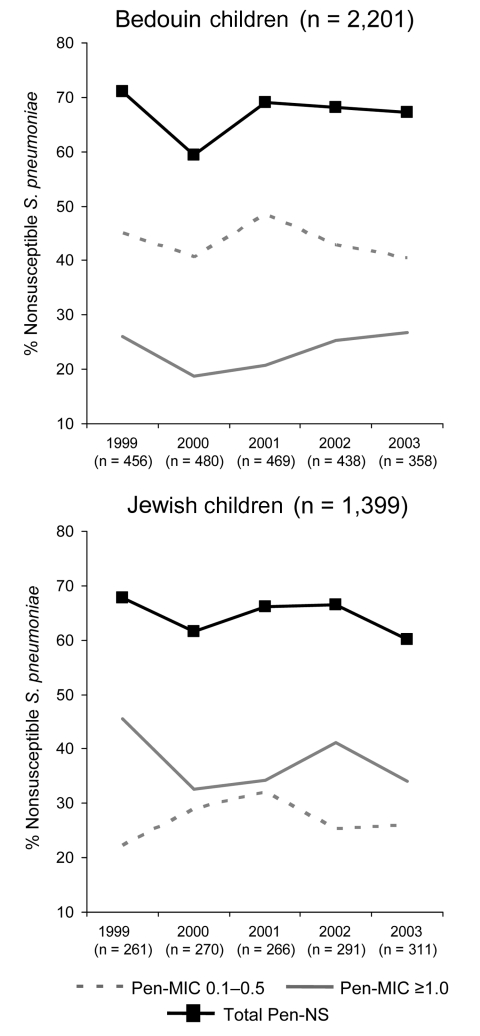
Proportions of penicillin-resistant *Streptococcus pneumoniae* isolated during episodes of acute otitis media in Bedouin and Jewish children <5 years of age in southern Israel from 1999 through 2003. Pen-MIC, penicillin MIC (μg/mL); Pen-NS, penicillin-nonsusceptible.

A total of 98% (3,600/3,651) of *S. pneumoniae* isolates were available for antimicrobial susceptibility testing. Penicillin-nonsusceptible isolates constituted most of the isolates (62% [869/1,399] and 64% [1,418/2,201] of all isolates in Jewish and Bedouin children, respectively) without a significant change in time or in the relative proportions between the 2 populations (Figure 2). However, resistance patterns differed significantly. Among Jewish children, 52%–67% of all penicillin-nonsusceptible isolates had a penicillin MIC ≥1.0 μg/mL each year (532 of all 1,399 isolates, 38%). Among Bedouin children, only 30%–40% of the nonsusceptible isolates (528 of all 2,201 isolates, 24%) had a penicillin MIC ≥1.0 μg/mL each year (p<0.001). Thus, despite the reduction in the prescription rates of penicillins in both populations, no parallel decrease in penicillin nonsusceptibility was observed. Furthermore, the highest penicillin MIC values were continuously observed in Jewish children, for whom the lowest penicillin prescription rates were recorded.

**Figure 3:**
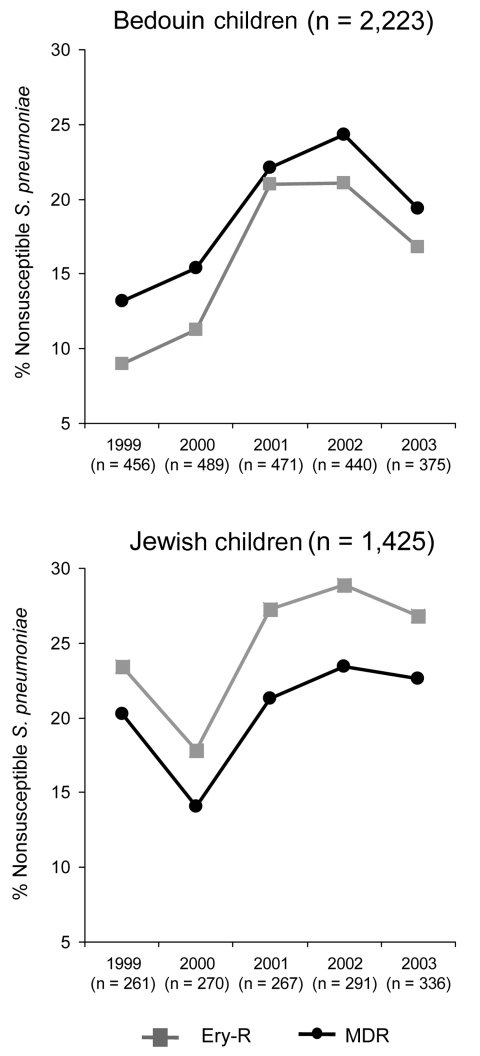
Proportions of erythromycin-resistant and multidrug-resistant *Streptococcus pneumoniae* isolated during episodes of acute otitis media in Bedouin and Jewish children <5 years of age in southern Israel from 1999 through 2003. Ery-R, erythromycin resistance; MDR, multidrug resistance (resistance to ≥3 antimicrobial classes).

Erythromycin resistance was more common in Jewish (25%, 356/1,425) than in Bedouin children (16%, 355/2,221) (p<0.001). In Bedouin children, rates of erythromycin resistance increased significantly from 1999 through 2002, from 9% (41/456) to 21% (93/440), respectively (p<0.001). In Jewish children, erythromycin resistance decreased from 23% (61/261) in 1999 to 18% (48/270) in 2000 (p = 0.01). Thereafter, the proportion of resistant isolates increased as well, reaching 29% (84/291) in 2002 (p = 0.02). Erythromycin resistance did not increase in either population during 2003. In 2003, 17% (63/375) and 27% (90/336) of isolates in Bedouin and Jewish children, respectively, were erythromycin-resistant (Figure 3). The multidrug-resistance pattern paralleled that of erythromycin resistance in each population. Thus, both erythromycin and multidrug resistance increased in parallel with the increase in the azithromycin prescription rate, and resistance tended to decrease with the change in this rate. Furthermore, the population with the highest azithromycin prescription rate also had the highest rates of erythromycin resistance.

**Figure 4:**
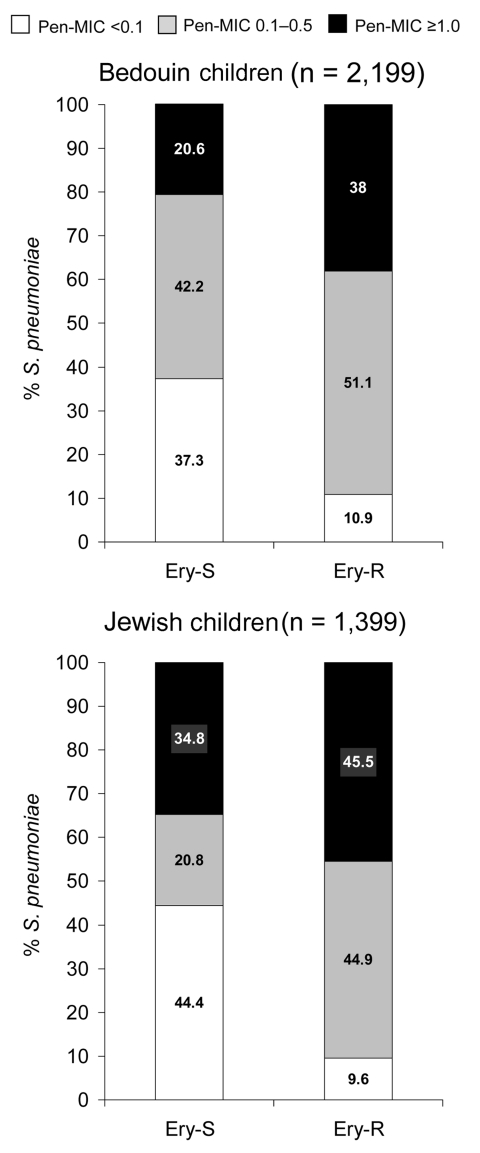
Distribution of penicillin MICs (μg/mL) in erythromycin-susceptible and erythromycin-resistant *Streptococcus pneumoniae* isolated during episodes of acute otitis media in Bedouin and Jewish children <5 years of age in southern Israel from 1999 through 2003. P was calculated for the difference in overall distribution of penicillin MICs between erythromycin-susceptible and erythromycin-resistant isolates, as well as for difference in relative contribution of isolates with MICs ≥1.0 μg/mL to both groups (p<0.001, Ery-S versus Ery-R in both Bedouin and Jewish children). Ery-S, erythromycin susceptible; Ery-R, erythromycin resistant; Pen-MIC, penicillin MIC.

Penicillin MIC values were not equally distributed among erythromycin-susceptible and erythromycin-resistant *S. pneumoniae* isolates (Figure 4). In both Jewish and Bedouin children, the proportion of penicillin-susceptible *S. pneumoniae* was greater in erythromycin-susceptible isolates, and the proportion of isolates with a penicillin MIC ≥1.0 μg/mL was greater in erythromycin-resistant *S. pneumoniae* (p<0.001 for each comparison).

## Discussion

We attempted to determine whether antimicrobial prescription patterns were associated with resistance patterns among *S. pneumoniae* isolates from young children with acute otitis media in 2 populations treated by the same medical system, but living in separate communities with different lifestyles. We used prescription rates of ≈20% of the children <5 years of age in each population and a large collection of middle ear fluid *S. pneumoniae* isolates obtained from these 2 populations during the same time period. This unique opportunity enabled us to not only relate resistance dynamics to prescriptions dynamics for each population, but also to observe whether differences in antimicrobial prescription rates between populations could explain some of the differences in antimicrobial resistance patterns.

The association of azithromycin prescriptions with antimicrobial resistance patterns among *S. pneumoniae* isolated from middle ear fluid is noteworthy for several reasons. First, the prescription rate for azithromycin was the only one that was higher among Jewish children than among Bedouin children. Second, the azithromycin prescription rate pattern closely paralleled both macrolide and multidrug resistance in each population. Third, higher penicillin MIC values were associated with macrolide resistance, which explained, at least in part, the higher rates of *S. pneumoniae* isolates from middle ear fluid with a penicillin MIC ≥1.0 μg/mL among Jewish children who received relatively fewer amoxicillin (with or without clavulanate) prescriptions than Bedouin children who received more amoxicillin prescriptions (with or without clavulanate).

The distinct pattern of reducing total antimicrobial drug prescriptions that resulted from reducing prescribed penicillins, although azithromycin use increased, was reported in other regions, including the United States (21,22) and western Europe (23). The pattern of reduced antimicrobial drug use could be the result of campaigns such as those conducted in the United States following the initiative by the Centers for Disease Control and Prevention for the judicious use of antimicrobial drugs, which recommended the first-line use of amoxicillin to treat acute otitis media (21) (http://www.cdc.gov/drugresistance/community/files/ads/Otitispa.pdf). However, the increase in azithromycin prescriptions, along with a reduction in penicillin prescriptions, could be partly the result of commercial promotion campaigns for the use of azithromycin, which were launched in parallel with campaigns to reduce the overall use of antimicrobial agents.

The increase in the azithromycin prescription rate in our study, as in Europe and North America, is partly attributable to the properties that make this drug an attractive agent for children. The long half-life of azithromycin (≤72 h) (24) makes a convenient dose regimen of once a day for ≤5 days. However, it is eliminated very slowly and remains at subinhibitory concentrations in tissues of persons with pneumococcal infections. Subinhibitory concentrations of antimicrobial agents favor the selection of resistant mutants. This has been shown in vitro for *S. pneumoniae* exposed to subinhibitory macrolide concentrations (25). In a clinical trial, children treated with azithromycin harbored significantly more resistant strains in their oral flora than those who randomly received other macrolides. After 6 weeks, 85% still had macrolide-resistant organisms (26). In European countries, the increase in prescriptions of long-acting macrolides resulted in selection for macrolide resistance in *S. pneumoniae* (27,28).

Our finding that azithromycin prescriptions were associated with *S. pneumoniae* multidrug resistance was noteworthy. The ability of certain antimicrobial agents to promote resistance to other drug classes has been previously reported. Several studies showed that use of long-acting macrolides was an important factor in increasing penicillin resistance in a given community (29,30). This could explain the higher rates of resistant *S. pneumoniae* with MIC≥1.0 μg/mL in Jewish than in Bedouin children, despite significantly lower prescription rates for penicillins, but significantly higher prescription rates for azithromycin. This pattern of increasing penicillin and macrolide resistance in association with increased prescribing of azithromycin was also observed in the United States, where it was predicted that in the absence of pneumococcal conjugate vaccine, by 2004 ≈40% of all *S. pneumoniae* isolates would be resistant to both penicillin and macrolides and that the increased rate would be exponential (31).

The differential effect of azithromycin versus amoxicillin on nasopharyngeal carriage of antimicrobial-resistant *S. pneumoniae* in patients was demonstrated in a study conducted in southern Israel. In this study, carriage of both macrolide- and multidrug-resistant *S. pneumoniae* markedly decreased in children with acute otitis media receiving amoxicillin-clavulanate, but increased markedly in those receiving azithromycin (32). This differential effect lasted more than 1 month.

Our study has 3 limitations. First, the factors contributing to differences in antimicrobial drug use between the 2 populations could not be controlled. Antimicrobial drug prescriptions could not be matched with individual use. In addition, potential confounders such as family structure and daycare exposure could not be assessed. The higher prescription rate for antimicrobial agents in Bedouin children could be explained by differences in accessibility to healthcare facilities. However, since there is no financial burden for healthcare in Israel and all clinics belong to the same health plan, acute otitis media is unlikely to be treated differently in either population. The similar reduction in antimicrobial drug prescriptions in both populations suggests that no difference in prescribing policies existed between the 2 populations. Lower socioeconomic status and overcrowding in the Bedouin population, which led to a higher rate of respiratory illness in this group (16,17), may explain the difference in rates of antimicrobial drug prescriptions.

Second, *S. pneumoniae* were obtained only from children with acute otitis media. However, *S. pneumoniae* is part of the normal nasopharyngeal flora and is exposed to antimicrobial agents regardless of the diagnosis for which the agent was prescribed. Therefore, we believe that the effect of prescribing antimicrobial drugs in the community on resistance patterns of *S. pneumoniae* isolated from middle ear fluid represents the effect on the entire spectrum of *S. pneumoniae* disease.

Third, this was not an intervention study; therefore, we could not demonstrate unequivocally the causative effect of azithromycin use on macrolide and multidrug resistance in *S. pneumoniae*. However, the association demonstrated in this study, together with published data, strongly suggest such a causative effect.

The introduction of the 7-valent pneumococcal conjugate vaccine to infant and toddler immunization programs in the United States was associated with a reduction in invasive diseases (33,34) and acute otitis media (35,36) caused by antibiotic-resistant *S. pneumoniae*. However, persistence of antimicrobial resistance within vaccine and nonvaccine serotypes (37–40) suggests that vaccine alone may not reduce antimicrobial resistance, and that if the use of antimicrobial drugs is not controlled, the ability of the pneumococcal conjugate vaccine to reduce antimicrobial-resistant S. *pneumoniae* may be only transient.

During the last 2 years of this study, prescription rates were reduced in both populations. This reduction could be partly explained by efforts of pediatric infectious diseases specialists to educate pediatricians and family physicians in the study area to reduce use of antimicrobial drugs, especially oral use of cephalosporins and macrolides-azalides. However, this effect may not be the main reason for this decrease. A decrease in macrolide and multidrug resistance of *S. pneumoniae* observed in the last year of this study may indicate that the effect of azithromycin use on antimicrobial resistance is reversible. Continuous monitoring of antimicrobial prescriptions and resistance in respiratory pathogens should help determine if a further decrease in azithromycin prescriptions would be followed by a further decrease in antimicrobial resistance of *S. pneumoniae*.

In conclusion, azithromycin prescriptions were associated with macrolide, penicillin, and multidrug resistance among *S. pneumoniae* isolated from the middle ear fluid of children with acute otitis media. Such an association was not found with amoxicillin (with or without clavulanate) prescriptions. When promoting judicious use of antimicrobial drugs, selective reduction in prescribing specific antimicrobial drugs such as azithromycin should be emphasized, in addition to total reduction in antimicrobial use.
